# Species-Specific Non-Physical Interference Competition among Mosquito Larvae

**DOI:** 10.1371/journal.pone.0088650

**Published:** 2014-02-18

**Authors:** Alon Silberbush, Ido Tsurim, Ran Rosen, Yoel Margalith, Ofer Ovadia

**Affiliations:** 1 Center for Biological Control (CBC), Department of Life Sciences, Ben-Gurion University of the Negev, Beer Sheva, Israel; 2 Department of Life Sciences, Achva Academic College, Arugot, Israel; 3 Department of Entomology, Agricultural Research Organization, The Volcani Center, Bet Dagan, Israel; Swedish University of Agricultural Sciences, Sweden

## Abstract

Individuals of different sex, size or developmental stage can compete differently and hence contribute distinctively to population dynamics. In species with complex life cycles such as insects, competitive ability is often positively correlated with larval developmental stage. Yet, little is known on how the development and survival of early-instars is influenced by interference from late-instar larvae, especially at low densities when exploitative competition is expected to be negligible. Furthermore, the specificity and mechanisms by which interference competition operates are largely unknown. We performed two complementary experiments aiming to quantify the competitive effects of late instar *Ochlerotatus caspius* on early instar larvae at low densities and under high resource supply rate. The first experiment examined the net effect of interference by 4^th^ on 1^st^ instar *O. caspius* larvae, relative to the effect of 1^st^ instars on themselves. The second experiment examined the effect of species-specific, non-physical interference competition (i.e., cage larvae) by 4^th^ on 1^st^ instar *O. caspius* larvae at low or high densities. Specifically, we compared the responses of *O. caspius* larvae raised in the presence of caged con- or hetero-specific, *Culiseta longiareolata*, with that of larvae in the empty-cage control group. As expected, interference from late instar larvae had a net negative effect on the development rate of first instars. In contrast, the presence of caged con-specifics (non-physical interference) accelerated the development rate of *O. caspius*, however, this pattern was only evident at the low density. Notably, no such pattern was detected in the presence of caged hetero-specifics. These results strongly suggest the existence of species-specific growth regulating semiochemicals.

## Introduction

Understanding the mechanisms that control population dynamics is essential for predicting ecological, economic, and health impacts of resident and invasive species [1]–[4]. At the heart of these mechanisms are density-dependent competitive effects on survival, development and reproduction [5]–[9]. Historically, ecologists distinguished between direct and indirect types of competition. Interference competition occurs through physical (e.g., aggression) or non-physical (e.g., chemical) interactions, resulting in direct negative effect on competitors fitness. In contrast, exploitation competition occurs when a shared limited resource is depleted, and consequently fitness is indirectly reduced [10]–[11]. A growing body of literature illustrates the importance of both exploitative and interference competition in triggering density- and frequency-dependent responses that can alter population and community dynamics [11]–[14]. Disentangling the negative effects of exploitative and interference competition requires preventing physical contact (e.g., caged competitors) [15], or creating a competitor-conditioned environment [16]. In this study, we adopted a similar approach to distinguish between physical and non-physical interference competition.

In organisms with complex life cycles, such as insects and amphibians who alter their habitat, diet and morphology over time, competitive ability can be positively correlated with developmental stage, meaning that the negative competitive effects on survival and life history traits should be stronger among larvae at early developmental stages [3], [17]–[20]. Such structural differences in competitive abilities can play an important role in determining population dynamics [21]–[24].

Mosquito larvae often occur at high densities, consequently experiencing both exploitative and interference competition. Hetero- and con-specific density-dependence among larval mosquitoes can strongly affect larvae and adult life history traits and fitness [3]. Increased larval densities negatively affect a range of life history traits including prolonged (delayed) larval development, reduced larval and adult survival, modified dispersal behavior and capabilities, and reduced female fecundity [25]–[33]. Such effects are largely the result of exploitative and interference competition caused by food and nutrient shortage, modified foraging behavior, and exposure to chemicals, waste products or other growth retardant factors [3], [34]–[35]. Structural differences in competitive abilities associated with larval size or developmental stage can interact with these factors to affect population and community dynamics [4], [18], [36]–[37].

First instar larvae of the tree hole mosquito *Aedes sierrensis* Ludlow, 1905exposed to chemicals produced by fourth instars have been shown to have a longer developmental time and smaller body size as adults [38]. However, these patterns were evident only at relatively high densities. In this study, we examined the potential effects of physical (i.e., free ranging larvae) versus non-physical (i.e., caged larvae) interference by a small number of late instars (4^th^ stage) on the development and survival of early instar larval mosquitoes *Ochlerotatus caspius* Pallas, 1771 (Diptera: Culicidae). We hypothesized that early instar larvae should be negatively affected by non-physical interference competition from the late instars. We further posited that these effects are likely species-specific. To test this second hypothesis, we used hetero-specific competition from *Culiseta longiareolata* Macquart, 1838 as control. *Culiseta longiareolata* populations overlap with *O. caspius* over a large geographic area [39]–[44]. Larvae of both species often co-occur in small temporary pools where the larger *C. longiareolata* larvae are superior competitors [45]. In addition, late instar *C. longiareolata* larvae exhibit strong interference competition against other species inhabiting the pool [44], [46]–[47]. We hypothesized that in shared habitats, larval *C. longiareolata* should negatively affect various aspects of *O. caspius* life history, including larval survival, development rate, adult size, and possibly also adult fecundity and sex ratio. We predicted that the development rate, survival, and resulting adult size of early instars will be negatively affected by the presence of late instar larvae. Similar effects are expected also under non-physical interference competition. If these effects are species-specific, they should vary between caged fourth instar *O. caspius* and *C. longiareolata*.

## Materials and Methods

Ethics Statement: No specific permits were required for the described field collections and lab studies. We performed two complementary experiments. The first experiment examined the net effect of interference competition (i.e., physical and non-physical) by 4^th^ on 1^st^ instar *O. caspius* larvae. The second experiment examined the effect of non-physical interference competition (i.e., cage larvae) by 4^th^ on 1^st^ instar *O. caspius* larvae. To test for species-specific effects, we contrasted the responses of 1^st^ instar *O. caspius* to caged con- or hetero-specific (*C. longiareolata*) 4^th^ instar larvae.

We used round plastic cups (8 cm diameter; 11.5 cm height), filled with 400 ml water as microcosms in the two experiments. The cups containing the larvae were placed at random order, in a temperature controlled room. Photoperiod was 13:11 hours light: dark, and room temperature was 28±2°C. Larvae were fed a finely grind mixture of “Sera pond” bio-flakes and “Kopoleh” rodent chow (21.4% protein). Surface scum was removed, to prevent larvae asphyxiation, and evaporated water refilled daily. We assumed that daily formation of surface scum indicates overfeeding.

*O. caspius* eggs originated from a colony established from individuals taken from the Dead-Sea area over the previous year. The eggs were hatched 24 hours prior to the experiment. *C. longiareolata* larvae originated from 11 egg rafts collected in the area of Beer-Sheva, Israel. In both experiments, the experimental cups were filled using a single-source tap water aged for 24 hours.

Previous work with *Aedes* and *Ochlerotatus* larvae suggest that density-dependent effects on survival either begin at 50 larvae per 400 ml (8 ml per larva), or that this density is considered low compared to natural populations [32], [48]–[52]. We therefore used larval densities lower than this threshold, aiming to reduce crowding effects to a minimum. Larvae were overfed, reducing competition for food to a minimum.

### Experiment 1 – Net effect of interference competition

Larvae were allocated into two treatments:

1. *Homogeneous*: stocking 40 1^st^ larvae in a cup.
2. *Heterogeneous*: stocking 30 1^st^ and 10 4^th^ instar larvae in each cup.

To maintain a constant density of 10 4^th^ instar larvae per cup during the first 5 days of the experiment (until the stocked 1^st^ instars reached 3^rd^ instar stage), pupating 4^th^ instar larvae were replaced with fresh ones. On day six, all stocked 4^th^instar larvae were removed from the cups, so that we could continue monitoring the development of the 30 stocked 1^st^ instar larvae. Starting from day seven, we daily removed all pupating larvae of this latter group from the cups and placed them in separate emergence vials, for sex determination and size measurements. Larvae were fed 0.06 grams food every 6 days, beginning day zero. This amount of food is considered overfeeding for larval densities used in the current study [51]. The daily accumulation of surface scum observed in our experiments is also indicative of overfeeding. Each of the two treatments was replicated 11 times; however 4 replicates of the heterogeneous treatment were excluded from the analysis owing to sudden extensive larval mortality.

### Experiment 2 – Non-physical interference competition

First instar *O. caspius* larvae were stocked into the experimental cups at densities of either10 or 25 larvae per cup and were subjected to one of the following treatments:

1. *Caged con-specifics:* Each experimental cup contained two caged 4^th^ instar larvae.
2. *Caged hetero-specifics:* Each experimental cup contained two caged 4^th^ instar *C. longiareolata* larvae (a superior hetero-specific competitor).
3. *Empty-cage control:* Experimental cups contained an empty cage.

In order to minimize the possible effect of exploitative competition, we used here a smaller number of larvae than in the first experiment. In addition, the larvae in the experimental cups were fed 0.06 grams every 3 days (double the amount provided during the first experiment). The caged larvae were maintained in a cylindrical, 3 cm diameter cages of fine mesh net (0.2×0.2 mm holes rolled twice so that 1^st^ instar larvae were not able to pass). The cage, containing two 4^th^ instar larvae, was placed at the center of each cup. Pupated or dead larvae were replaced daily until the experiment was terminated. Water in cups was stirred daily to increase the exchange of water between the cage and surrounding water. Each treatment×density combination was repeated 7 times (3 treatments×2 densities×7 replicates = 42 experimental cups in total).

As in Experiment-1, we daily removed all pupating larvae from the water surrounding the cages and placed them in separate emergence vials, for sex determination and size measurements.

### Data analysis

To evaluate the effects of our experimental manipulation on mosquito life history, we measured the overall larval survival, emerging adults' sex ratio, wing length, and larval development time.

#### Larval Survival

Since we could not determine the exact mortality time of each larvae, we could not use time-to-event analysis to analyze survival rates. Instead, we measured larval survival as the proportion of 1^st^ instar larvae surviving to adulthood (i.e., emerging as adults) per experimental cup. Because we could not determine larval sex at the onset of the experiment, larval survival was calculated for both sexes in the aggregate. Data were analyzed using ANOVA after applying an angular (arcsin[sqrt(p)]) transformation [53].

#### Sex ratio

We calculated sex ratio as the proportion of emerging females per cup. Data were analyzed using ANOVA after applying an angular (arcsin[sqrt(p)]) transformation [53].

#### Wing length

After emergence, adults were labeled and kept at −30°C for subsequent measurements. Adults were then dried and their wings removed and placed on a microscope slide with an embedded scale. The wings were then photographed using Dino-Lite digital microscope (model AM-413T) at 1.3 megapixels and measured to the nearest 10^−3^ mm using ImageJ 1.43u [54]. Wings were measured from the axillary incision to the wing tip. As in other mosquito species, *O. caspius* wing length correlates with adult's weight and fitness [55]. Individual average wing length was used as the response variable. To avoid pseudo-replications individuals were nested within experimental cups, i.e., cups rather than individuals were used as replicates. In experiment 1, we used nested ANOVA to test for the effect of treatment (homogeneous vs. heterogeneous) on wing length. Practically, in the statistical model experimental cup ID was nested within treatment. In experiment 2, we used nested ANOVA to test for the effect of treatment (caged con-specifics, caged hetero-specifics, empty-cage control) and initial density on wing length. Practically, in the statistical model, experimental cup ID was nested within Treatment×Density interaction.

#### Larval development time

Larval development time was measured as the number of days from hatching to pupation. We used Cox proportional hazards model to analyze the effect of treatment (experiment 1), and treatment and density (experiment 2) on larval pupation rate (development time of individual larvae clustered per replicate). This method allows the evaluation of effects of different predictors on occurrence rate (i.e., larval pupation), independent of the time varying background pupation rate [56]. Using the Cox proportional hazards model allowed us to estimate a coefficient (β) for each one of the predictor variables and test for its significance. The exponent coefficient (e^β^), estimates the expected change in the event occurrence rate per one unit change in the covariate. For instance, e^β^ = 0.5 for initial density means that the addition of a single competitor larva will result in halving pupation rate (i.e., lengthening of larval development time), averaged over the entire experiment duration. The significance level of each coefficient is determined using Z statistic, calculated as the ratio β/SE(β). The overall significance level of the model is determined using Wald test, examining the null hypothesis that all β's in the model equal to zero. To avoid pseudo-replications and to account for possible correlation between individuals within each experimental cup, we used a robust jackknife variance estimator grouped by observations larvae per cup [57]. In our analyses, larval development time (from hatching to pupation) was the response variable. This analysis was performed on the total number of larvae finally emerging as adults, so that sex could be determined accurately. In experiment 1, treatment was coded as a binary explanatory variable (heterogeneous = 0 and homogeneous = 1). In experiment 2, treatment was converted into a dummy variable while using the empty-cage control as the reference group (caged con-specifics *O. caspius* = 1, *C. longiareolata* = 0; caged hetero-specifics *O. caspius* = 0, *C. longiareolata* = 1; empty-cage control *O. caspius* = 0, *C. longiareolata* = 0). In other words, con- and hetero-specific treatments were contrasted with the empty-cage control group. As for initial density, we used the actual number of individuals stocked into the experimental cups.

## Results

### Larval survival

#### Experiment 1 - Net effect of interference competition

Survival of 1^st^ instar larvae was not affected by the presence of con-specific 4^th^ instar larvae (F_1,17_ = 0.02, P = 0.89; Average proportion of surviving individuals ±1S.D: 0.2±0.12 and 0.19±0.11 for the homogeneous and heterogeneous treatments, respectively).

#### Experiment 2 – Non-physical interference competition

At the high density, survival of 1^st^ instar larvae tended to be higher in the empty-cage control group compared to the other two treatments. However, the interaction between treatment and initial density was not significant (F_2,36_ = 1.82, P = 0.177), nor were there significant main effects (Treatment: F_2,36_ = 1.48, P = 0.242, Initial density: F_1,36_ = 3.93, P = 0.055).

### Sex ratio

Females comprised 47% and 45% of total emerging adults in the first and second experiments, respectively. None of the main effects or their respective interaction terms were statistically significant (Experiment 1: F_1,17_ = 2.32, P = 0.15. Experiment 2: Density: F_1,36_ = 0.054, P = 0.818, Treatment: F_2,36_ = 0.733, P = 0.487, Treatment×Density: F_2,36_ = 0.007, P = 0.802).

### Time to Pupation

#### Experiment 1 - Net effect of interference competition

**Figure 1:**
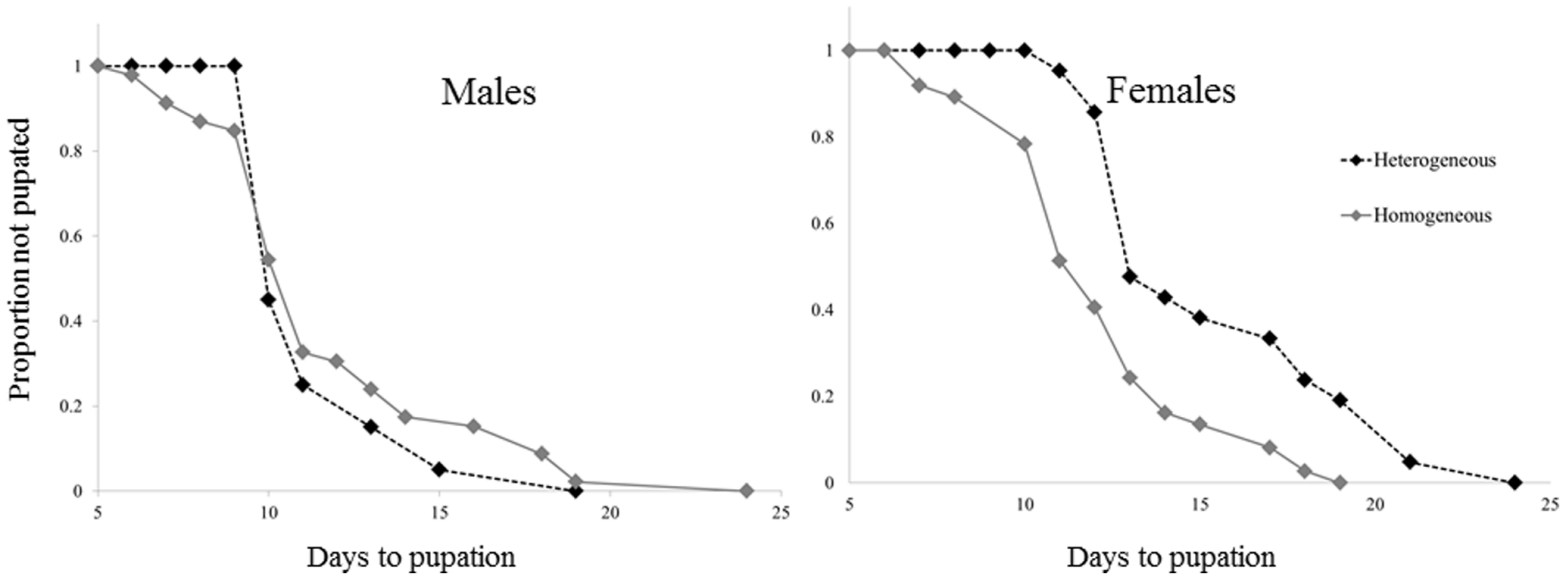
A Kaplan-Meier fit for the relationship between the time since hatching and the proportion of larvae not yet pupated (out of the final emerging population). Female larvae developing in the Homogeneous-stage treatment developed faster than those developing in the Heterogeneous stage treatment (in the presence of 4^th^ instar larvae). Males were not affected by treatment.

Analysis using Cox proportional hazards model indicated that the presence of 4^th^ instar larvae caused a ∼2.6 fold reduction in the female pupation rate (P<<0.001, Table 1; Fig. 1), while having no significant effect on the pupation rate of males (P = 0.528, Table 1; Fig. 1).

**Table 1:** The effect of Treatment (Heterogeneous vs. Homogeneous stage) on the development rate of 1^st^ instar larvae to pupation: Results of Cox proportional hazards model for the effect of treatment on the rate of larval pupation.

Sex	Regression Coefficient (β)	e^β^	SE(β)	Z	n	P	Wald test
Females	0.97	2.64	0.289	3.356	58	<<0.001	**Wald = 11.3 P<<0.001 df = 1**
Males	−0.193	0.825	0.305	−0.633	66	0.53	**Wald = 0.4 P = 0.528 df = 1**

For the analysis, treatments were coded as Heterogeneous = 0, Homogeneous = 1. Higher pupation rate means shorter development time. Analysis was performed on the two sexes separately.

#### Experiment 2 – Non-physical interference competition

**Figure 2:**
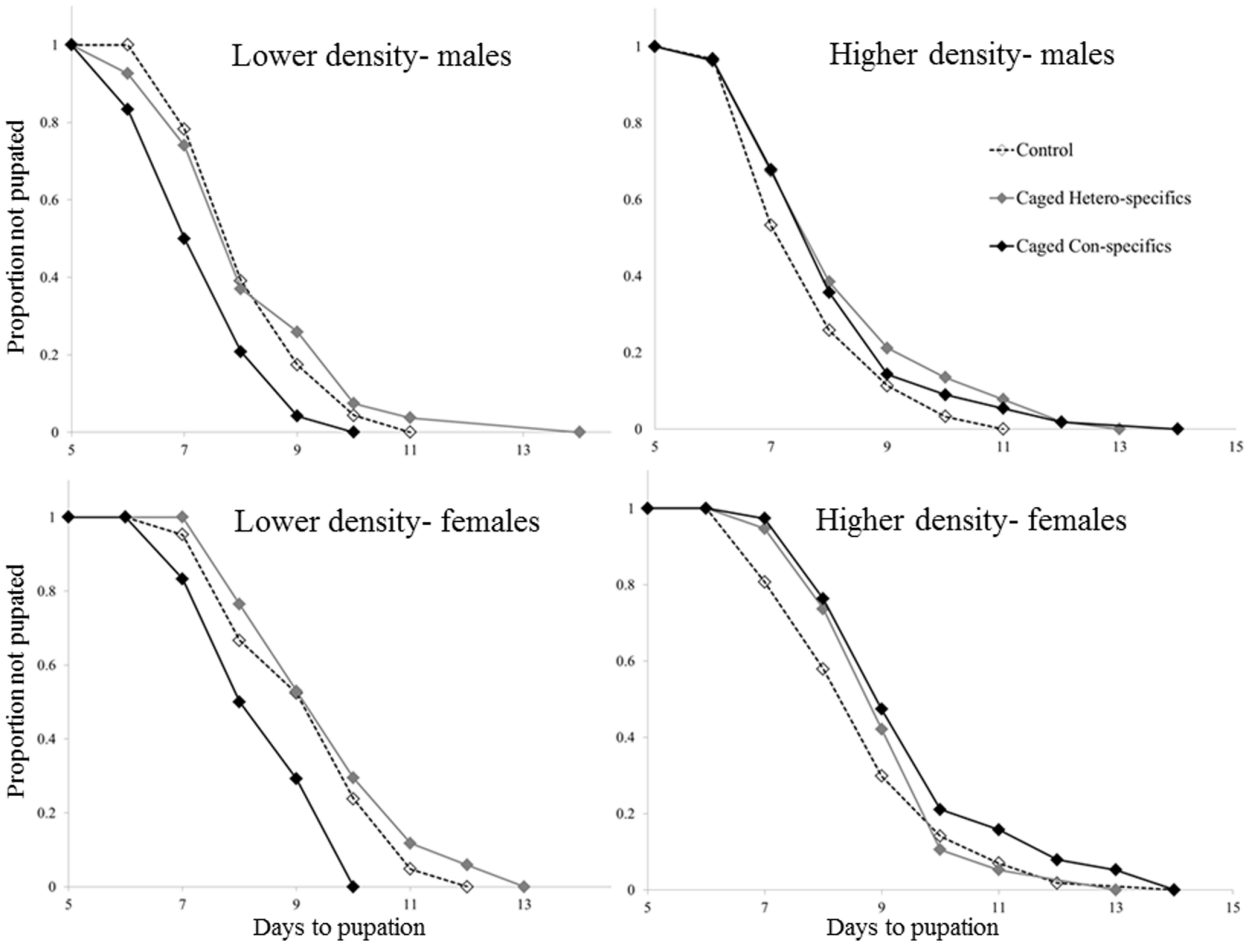
A Kaplan-Meier fit for the relationship between the time since hatching and the proportion of larvae not yet pupated (out of the final emerging population). In the lower initial density, both males and females pupated sooner in the Caged-conspecific treatment (caged *O. caspius* 4^th^ instar larvae); relative to the Empty-cage control treatment. This pattern was not evident in the higher initial larval density. Moreover, caged-heterospecifc treatment (caged *C. longiareolata* 4^th^ instar larvae) did not affect larval pupation rate, relative to control.

The presence of caged con-specifics brought about a ∼3.7 fold increase in female pupation rate, relative to the empty-cage control (P = 0.006, Table 2). However, this pattern was only evident at the low density (Caged con-specifics×Density interaction, P = 0.004; Table 2; Fig. 2). We could not detect similar patterns in the caged hetero-specifics treatment (Caged hetero-specifics: P = 0.510; Caged hetero-specifics×Density interaction: Z = 0.077, P = 0.940; Fig. 2). Similarly, the presence of caged con-specifics caused a ∼2.5 fold increase in male pupation rate, relative to the empty-cage control (P = 0.043; Table 2; Fig. 2). Here too, this pattern was only evident at the low density (Caged con-specifics×Density interaction, P = 0.043; Table 2; Fig. 2). Again, we could not detect similar patterns in the caged hetero-specifics treatment (Caged hetero-specifics: P = 0.160; Caged hetero-specifics×Density interaction: Z = −0.693, P = 0.490; Fig. 2).

**Table 2:** The effect of Treatment^a^, on the development rate of 1^st^ instar larvae to pupation.

Sex	Variable	Regression Coefficient (β)	e^β^	SE(β)	Z	P	Wald test
Females	Density	0.018	1.018	0.014	1.283	0.2	**Wald = 8.79; df = 4 n = 195; P = 0.067**
	Caged Con-specifics	1.312	3.712	0.476	2.754	0.006	
	Caged Hetero-specifics	−0.151	0.86	0.231	−0.656	0.51	
	Density×Caged Con-specifics	−0.07	0.933	0.024	−2.884	0.004	
Males	Density	0.011	1.011	0.014	0.79	0.43	**Wald = 8.36; df = 4; n = 244; P = 0.079**
	Caged Con-specifics	0.915	2.498	0.451	2.028	0.043	
	Caged Hetero-specifics	−0.334	0.716	0.237	−1.411	0.16	
	Density×Caged Con-specifics	−0.048	0.953	0.024	−2.025	0.043	

Results of Cox proportional hazards model for the effect of treatment on the rate of larval pupation. Higher pupation rate means shorter development time. Analysis was performed on the two sexes separately.

### Wing length

#### Experiment 1 - Net effect of interference competition

The presence of 4^th^ instar larvae had no significant effect on the wing length of females (Table 3) or males (Table 4).

**Table 3:** The effect of Treatment (Homogeneous vs. Heterogeneous stage), on the wing length of females emerging from the experimental cups.

	SS	Df	MS	F	P
**Treatment**	0.014	1	0.014	0.158	0.696
**Cup(Treatment)**	1.355	15	0.090	2.825	0.004
**Error**	1.311	41	0.032		

Nested ANOVA testing for the effect of treatment on female wing length. Individuals are nested within experimental cups, i.e., experimental cups rather than individuals are considered as the replicates. Practically, in the statistical model, experimental cup ID was nested within treatment. Note that the MS of this nested variable was used as the error term of treatment.

**Table 4:** The effect of Treatment (Homogeneous vs. Heterogeneous stage), on the wing length of males emerging from the experimental cups.

	SS	df	MS	F	P
**Treatment**	0.002	1	0.002	0.022	0.885
**Cup(Treatment)**	1.431	14	0.102	3.676	<0.001
**Error**	1.332	48	0.028		

Nested ANOVA testing for the effect of treatment on male wing length. Individuals are nested within experimental cups, i.e., experimental cups rather than individuals are considered as the replicates. Practically, in the statistical model, experimental cup ID was nested within treatment. Note that the MS of this nested variable was used as the error term of treatment.

#### Experiment 2 – non-physical interference competition

Increased density resulted in a significant reduction in the wing length of females (Table 5), but no such effect was evident among the males (Table 6). Both the effect of treatment, and density×treatment interaction were not significant (Tables 5 and 6 for females and males, respectively).

**Table 5:** The effect of Density and Treatment (caged con-specifics, caged hetero-specifics, empty-cage control) on the wing length of females emerging from the experimental cups.

	SS	df	MS	F	P
**Treatment**	0.013	2	0.006	0.143	0.867
**Density**	0.274	1	0.274	6.524	0.015
**Density×Treatment**	0.036	2	0.018	0.429	0.655
**Cup(Density×Treatment)**	1.462	35	0.042	2.33	<0.001
**Error**	2.649	146	0.018		

Nested ANOVA testing for the effect of treatment and initial density on female wing length. Individuals are nested within experimental cups, i.e., experimental cups rather than individuals are considered as replicates. Practically, in the statistical model experimental cup ID was nested within Treatment×Density interaction. Note that the MS of this nested variable was used as the error term of treatment, density and Treatment×Density interaction.

**Table 6:** The effect of Density and Treatment (caged con-specifics, caged hetero-specifics, empty-cage control) on the wing length of males emerging from the experimental cups.

	SS	df	MS	F	P
**Treatment**	0.071	2	0.036	0.947	0.397
**Density**	0.003	1	0.003	0.079	0.780
**Density×Treatment**	0.003	2	0.001	0.026	0.974
**Cup(Density×Treatment)**	1.358	36	0.038	1.226	0.193
**Error**	5.874	189	0.031		

Nested ANOVA testing for the effect of treatment and initial density on male wing length. Individuals are nested within experimental cups, i.e., experimental cups rather than individuals are considered as replicates. Practically, in the statistical model experimental cup ID was nested within Treatment×Density interaction. Note that the MS of this nested variable was used as the error term of treatment, density and Treatment×Density interaction.

## Discussion

Variation in competitive abilities among different developmental stages within a population can play an important role in determining population dynamics [21]–[24]. Our results indicate that interference competition of 4^th^ on 1^st^ instar larval *O. caspius* have significant effects on development rate, even when exploitative competition should be negligible. Net con-specific interference from 4^th^ instar larvae caused significant reduction in female pupation rate (longer development time), but no such effect was detected among males. Non-physical interference from caged con-specific 4^th^ instar larvae resulted in increased pupation rate of both female and male *O. caspius* (shorter development time); however, this effect was evident only at low larval densities. Interestingly, no such effect was detected in the presence of caged 4^th^ instar *C. longiareolata* larvae – a superior hetero-specific competitor – implying a species-specific non-physical interference competition.

Negative density-dependence can shift adult sex ratio, while reducing larval survival [52] and adult size. Indeed, increased density resulted in a reduction in the wing length of female *O. caspius*. Consistent with our previous findings [45], adult male size of *O. caspius* was not affected by larval density. Additionally, larval density had no detectable effects on sex ratio or survival. These findings suggest that the larval densities used in this study were not high enough to strongly affect larval life history traits by themselves. Negative density-dependence in mosquitoes has been extensively reported and discussed [25]–[32], [48]–[52]. Part of these effects has been ascribed to chemicals possessing growth retardant effects, possibly excreted metabolic waste [31], [35], [38]. To the best of our knowledge, this is the first report of species-specific growth-related semiochemicals in mosquitoes. Our results lend further support to the importance of population structure and density-dependence for larval life history traits, even at densities considered well below carrying capacity for *Aedes* and *Ochlerotatus* [32], [48]–[52]. Other mosquito genera are known to respond to crowding at similar densities [58]–[59]. We are unaware of studies exploring the differences in density-dependence among mosquito genera. However, we speculate that open-habitat mosquitoes should be more sensitive to changes in larval densities as well as to related chemical cues than container species.

Larval resource allocation to the multitude of life history traits can be facultative [60]–[61], allowing some deferral of competitive effects on survival or other vital traits, at the expanse of investment in other, less vital ones. Male mosquitoes are likely subject to a stronger selection on shorter larval development time, and thus should be less conservative with respect to size or adult survival. Female mosquitoes require more investment in soma and reproductive organs, and probably experience weaker selection on larval developmental time, than on body size [62]. Indeed, in the first experiment, whose aim was to quantify net con-specific interference of 4th on 1st instar larval *O. caspius*, the developmental time of females increased significantly, while that of males remained the same. This extended development time probably allowed the females to accumulate all the required resources for proper development. In addition, owing to the delayed development, females could probably experience competitive release, when the more advanced stage larvae finally pupated. Interestingly, in the second experiment, the presence of caged con-specific 4^th^ instar larvae accelerated the development rate of both female and male larval *O. caspius*; however, this pattern was evident only at low density. In both experiments larval densities were below those known to have detectable negative density-dependence effects on several species of *Aedes* and *Ochlerotatus* larval survival [32], [48]–[51] including *O. caspius* (Tsurim I. unpublished data). Furthermore, both the high (25 larvae) and low (10 larvae) densities used in the second experiment were much lower than that of the first experiment (40 larvae). Indeed, qualitative comparison between the two experiments (homogeneous treatment of the 1^st^ experiment vs. empty cage controls of the 2^nd^ experiment) shows that development rate was substantially higher in the second experiment, suggesting that density reduction *per se* accelerates development rate. We suggest that at high density the presence of 4^th^ instar larvae causes a reduction in activity levels of early instar larvae and hence slower development rate. In contrast, at low densities, when perceived competition is low and accelerated development could be sustained energetically, the interference “signal” enhances development rate.

This study further exemplifies the complexity of density-dependent processes that regulate population dynamics, and demonstrate the potential importance of accounting for population structure, especially in organisms with a complex life cycle. Most importantly, this study probably provides the first indication of the potential role of species-specific chemical communication in regulating competition among larval mosquitoes. The exact nature of these chemicals, their evolutionary and ecological significance, and the mechanisms by which they operate to influence larval development are yet unclear to us, and should be the focus of future studies.
